# Tracking Chirality Evolution in Tellurium Nanocrystals Via Polarization-Resolved Second-Harmonic Scattering

**DOI:** 10.1021/acs.nanolett.6c01271

**Published:** 2026-06-15

**Authors:** Ruidong Ji, Bar Reuven, Bradleigh Kerrigan, Gil Markovich, Ventsislav K. Valev

**Affiliations:** † Department of Physics, 1555 University of Bath, Bath, BA2 7AY, U.K.; ‡ School of Chemistry, the Raymond and Beverly Sackler Faculty of Exact Sciences, 26745 Tel Aviv University, Tel Aviv 6997801, Israel; § Department of Electronic & Electrical Engineering, University of Bath, Bath, BA2 7AY, U.K.

**Keywords:** Chirality, chirality transfer, nanoparticles, nonlinear optics, chiroptical effects

## Abstract

Chirality pervades living systems and increasingly guides the design of functional nanomaterials. Yet weak or residual chirality can be difficult to detect, particularly in liquid-phase nanocrystal syntheses requiring noninvasive probes. Here we track chirality evolution in colloidal tellurium nanocrystals spanning strong, alloy-tuned, and reduced shape chirality. As morphological chirality decreases, linear circular dichroism drops by roughly an order of magnitude. In contrast, polarization-resolved second-harmonic scattering retains clear handedness-dependent responses. Across six independent observables—the nonlinear g-factor (g_NL_) and dual-circular polarization metrics (*DCP*_1_ and *DCP*_2_) measured in forward and right-angled geometries—the signal reverses sign between enantiomorphs and remains well-resolved. The geometry dependence of these responses is consistent with interference between mirror symmetry-even and mirror symmetry-odd nonlinear tensor contributions. These results establish chiroptical second-harmonic scattering as a sensitive probe of weak structural asymmetry in nanocrystals exhibiting coupled shape and crystal chirality.

## Graphical abstract

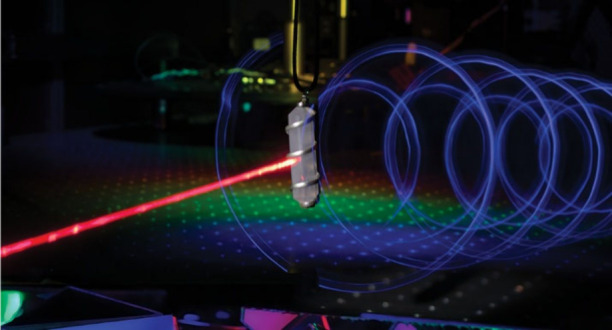

Chirality – the absence of mirror symmetry – plays a central role in chemistry, biology, and materials science,^1^ where structural asymmetry can propagate across multiple length scales.^2–5^ Synthetic nanomaterials increasingly follow analogous strategies. Chiral building blocks or chiral growth environments can bias nanoparticle shapes, assemblies, and optical responses.^6–10^ Strategies collectively described as *chirality transfer* ^11^ or *chirality conferral* ^12^ seek to propagate handedness from one structural level to another.^13,14^ For example, small chiral molecules have been shown to induce twisted morphologies in plasmonic gold^15–17^ and silver^18^ nanoparticles. In other systems, circularly polarized light has been used as a chiral field to bias the self-assembly.^19^ Similarly, chiral soft templates can direct the confined growth of inorganic nanocrystals.^20,21^ These chirality conferral mechanisms are actively pursued as routes to engineer materials with deliberately tailored chiroptical properties.^22^ Yet, just as the deliberate construction of chirality is important, so too can be its absence.

Strict polarization neutrality is required in a range of nanostructured optical technologies.^23,24^ Optical communication components rely on well-defined polarization states to minimize signal distortion;^25^ laser and spectroscopic systems require polarization purity for accurate measurements; LIDAR and interferometric metrology depend on stable polarization control; colloidal quantum dots^26^ and related nanocrystal luminophores are now widely integrated into display backlights, solid-state lighting, and emerging micro-LED architectures;^27–29^ and dielectric metasurfaces and metalenses are often engineered to operate independently of incident polarization.^30,31^ In such systems, unintended nanoscale chirality can introduce circular birefringence or circular dichroism that degrades device performance. Sensitive symmetry-resolved characterization methods are, therefore, critical for detecting residual structural asymmetry.

Nonlinear optical processes are highly sensitive to material symmetry that is expressed in the nonlinear susceptibility tensor elements. These elements contribute to the intensity and polarization of light detected at frequencies that are harmonics of the illumination light. Among the nonlinear optical effects most suitable for studying chiral inorganic nanoparticles in liquids is *chiroptical harmonic scattering*. Chiroptical harmonic scattering was first predicted in the 1970s.^32^ It was demonstrated in 2019, in plasmonic nanoparticles – silver nanohelices.^33^ Soon after, it was observed in gold helicoids,^34^ then in CdTe^35^ and in Si^36^ nanohelices. Alongside these experimental reports in chiral nanoparticles, the effect was observed in molecules, and numerous theoretical developments have also emerged.^37–40^ Yet in all previous studies, the light scatterers possessed a single type of chirality, expressed in their shape – Au, Ag, and Si are centrosymmetric, CdTe is not, but its crystal structure is achiral and the nanoparticles studied were polycrystalline with random arrangement of the crystallites. No chiroptical harmonic scattering studies have been performed on elemental tellurium (Te).

Te is a suitable system because the chirality of its nanocrystals is highly tunable.^41^ Te crystallizes in the chiral space groups *P*3_1_21 and *P*3_2_21, where atoms form helical chains along the *c*-axis.^42,43^ Te nanocrystals can also develop shape chirality through twisting and asymmetric faceting.^44^ Screw dislocations have been shown to generate such morphologies, and their handedness is not strictly dictated by the lattice enantiomer.^45^ As a result, shape chirality can be established, tuned, and reduced.^46^

Here, we investigate polarization-resolved second-harmonic scattering from colloidal Te nanocrystals whose morphological chirality is systematically tuned through growth conditions. This material platform allows us to follow chirality evolution across three regimes of structural asymmetry within a single nanocrystal system: strongly chiral morphologies, alloy-tuned nanocrystals with a modified optical response, and larger nanocrystals with substantially reduced shape chirality. Linear CD measurements reveal that the chiroptical response decreases markedly with this progression. In contrast, SH scattering retains clear handedness-dependent signatures across multiple polarization observables, including the nonlinear g factor and dual-circular polarization metrics (*DCP*_1_ and *DCP*_2_). Across all observables, left- and right-handed enantiomers produce opposite SH responses. The magnitude and sign of these observables depend strongly on the detection geometry, consistent with interference between dominant achiral and weaker chiral nonlinear tensor components. By tracking chirality evolution within a single colloidal material system, this work establishes nonlinear polarization-resolved scattering as a robust (across six observables) probe of weak structural asymmetry and extends chiroptical harmonic scattering to nanocrystals exhibiting both crystal and shape chirality.

Figure 1 shows the three sample classes. Te nanocrystals were synthesized by aqueous reduction of sodium tellurite with hydrazine in the presence of L- or D-penicillamine, which acts as a coordinating ligand, auxiliary reductant, and handedness-directing agent.^47,48^ Experimental details are provided in the Supporting Information. In principle, residual surface-bound penicillamine may also contribute to the local chiral environment on the nanocrystal surface. However, penicillamine is mainly active at shorter wavelengths (<275 nm), its surface coverage decreases as the crystals form, and its contribution is therefore expected to remain broadly comparable across the sample series and reflect the underlying lattice handedness.

**Figure 1:**
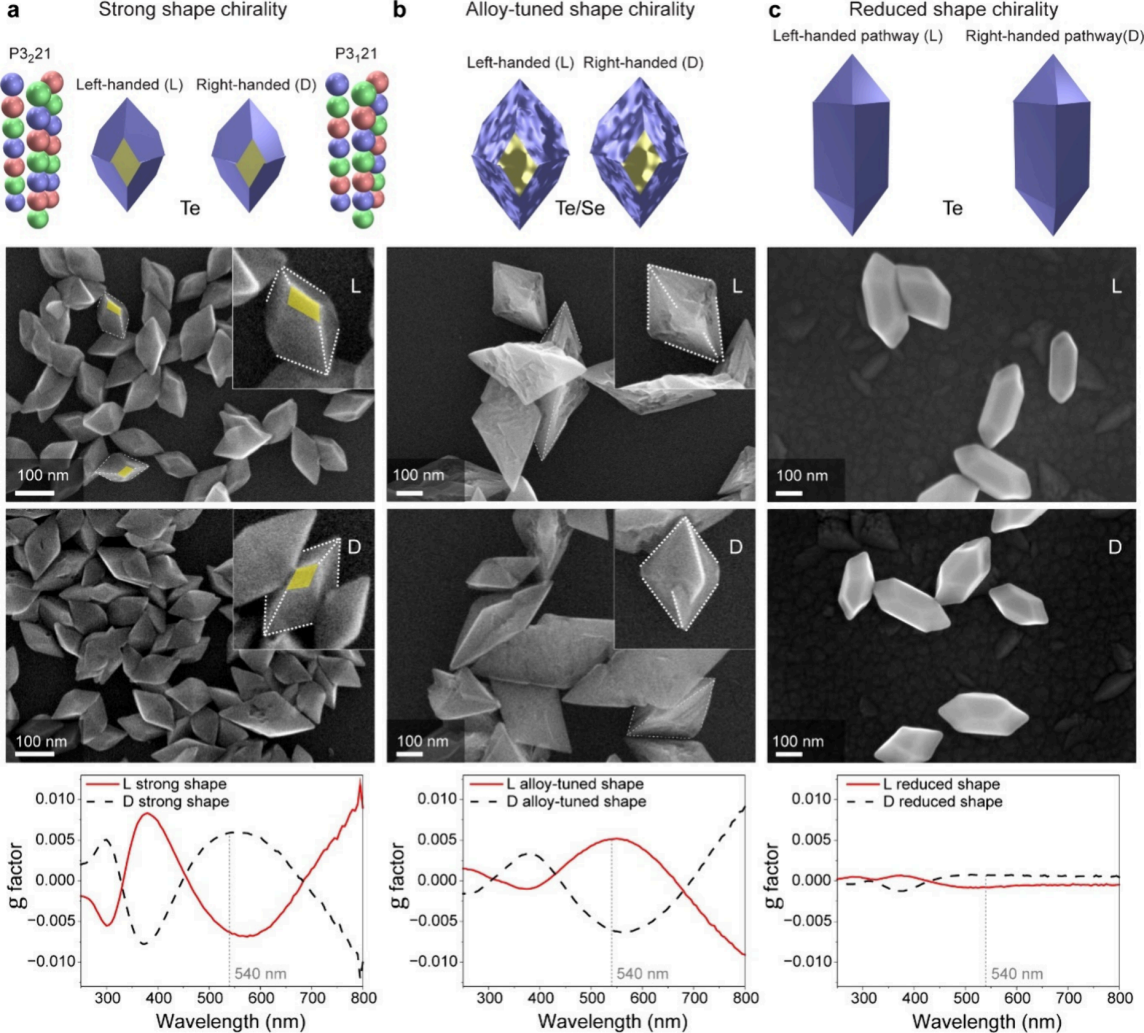
Evolution of crystal and shape chirality in Te nanocrystals. **a**, Te nanocrystals synthesized with L- or D-penicillamine adopt predominantly *P*3_2_21 and *P*3_1_21 crystal enantiomers, respectively, and exhibit strong shape chirality, as shown in scanning electron microscopy (SEM) images and highlighted facets (insets). Corresponding linear dissymmetry (g-factor) spectra display strong bisignate responses. **b**, Te/Se alloy nanocrystals prepared by introducing selenourea during growth retain pronounced shape chirality. Their g-factor spectra are red-shifted and spectrally modified relative to a. **c**, Prolonged growth and mild heating yield larger Te nanocrystals with reduced shape chirality and strongly suppressed g-factor magnitudes.

In Figure 1a, L- and D-shaped nanocrystals are illustrated in scanning electron micrographs (SEM). These nanocrystals were produced with L- and D-penicillamines, respectively. They adopt predominantly (80%) the *P*3_2_21 and *P*3_1_21 space groups, respectively.^49^ Beyond their crystal lattice chirality, the nanocrystals also develop strong shape chirality that can be seen in the SEM images. Correspondingly, they present strong, bisignate g-factor spectra in the linear optical regime.

In Figure 1b, the samples were obtained similarly to those in Figure 1a, with one key difference - the incorporation of selenium (Se). Immediately after adding hydrazine, a 0.1 M selenourea solution in dimethylformamide was introduced (35 μL, giving ∼ 3.5% nominal atomic Se content) to generate Te/Se alloy nanocrystals. The incorporation of Se again results in nanocrystals with pronounced shape chirality (as shown in the SEMs) and alloy-tunable chiroptical spectra (as shown by the g-factor plots that are significantly red-shifted compared to those in Figure 1a).

In Figure 1c, the reaction that produces the nanocrystals in Figure 1a was continued for ∼ 60 min, and after increasing the temperature to 40 °C, it was continued for an additional 3 h. This continuation results in larger nanocrystals (compared to those in Figure 1a), with reduced shape chirality, as illustrated by the SEM images. Their shape chirality reduction is also demonstrated by the g-factor spectra. The chirlaity of these nanocrystals was then investigated with second-harmonic (SH) scattering.

Figure 2a shows the SH scattering setup. Circularly polarized 1080 nm ultrafast pulses (200 fs, 80 MHz) were focused into 1 mL dispersions in a glass cuvette. SH light at 540 nm was collected in the forward (∥) and right-angled (⊥) geometries, filtered, and detected with a PMT and gated photon counter. Long-term power drift was corrected using a z-cut quartz reference.

**Figure 2:**
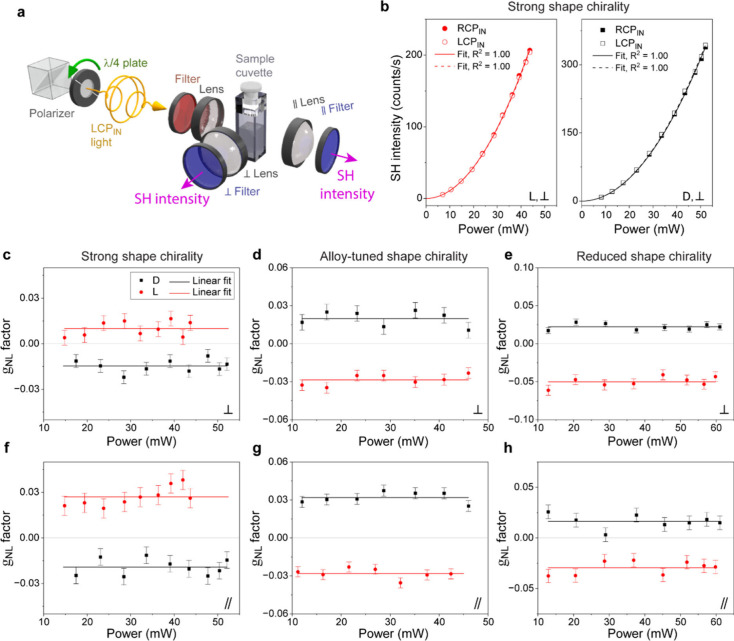
Robust second-harmonic (SH) chiroptical contrast despite reduced shape chirality. **a**, Schematic of the SH scattering experiment. SH light (540 nm) is collected in the forward (∥) and right-angled (⊥) geometries. **b**, SH intensity versus excitation power for Te nanocrystals with shape chirality measured in the ⊥ geometry. Filled and open symbols denote RCP_IN_ and LCP_IN_; solid lines are quadratic fits. **c–e**, Nonlinear dissymmetry factors g_NL_ versus excitation power in ⊥ scattering for nanocrystals with strong shape chirality (c), alloy-tuned chirality (d), and reduced chirality (e). **f–h**, g_NL_ measured in the ∥ geometry for the same sample classes. Opposite signs are observed for L and D enantiomorphs, and the nonlinear response is power-independent.

The graphs in Figure 2b correspond to the SH intensity as a function of the incident laser power for Te nanocrystals with a shape chirality. Data were collected in the right-angled geometry from both L- and D-shaped nanocrystals. The full and empty symbols indicate the RCP and LCP states of the incident light, respectively. In both enantiomorphs, the lines are excellent square law fits (R^2^ = 1), which is consistent with SH scattering.

The chiroptical response of the Te nanocrystals can be assessed by measuring the SH scattered intensity for RCP and LCP (*I*_*RCP*_^(2ω)^ and *I*_*LCP*_^(2ω)^, respectively) and then calculating the nonlinear g-factor (g_NL_) as

1 $$g_{\mathrm{NL}}=2\cdot \frac{I_{RCP}^{\left(2\omega \right)}-I_{LCP}^{\left(2\omega \right)}}{I_{RCP}^{\left(2\omega \right)}+I_{LCP}^{\left(2\omega \right)}}$$

In the present work, crystal and morphology-derived chirality are treated as jointly contributing to the measured nonlinear response. Figure 2c-e shows the g_NL_ as a function of illumination power at 1080 nm, in right-angled scattering. In each case, the g_NL_ obtained from the fits changes sign between the enantiomorphs. We note especially that in the case of reduced chirality g_NL_ remains substantial. Similarly, Figure 2f-h shows the g_NL_ measured in forward scattering. Again, the data are essentially constant as a function of illumination power, and all the enantiomorph pairs present opposite sign. Clearly, the obtained g_NL_ is again very pronounced in the case of the Te nanocrystals with reduced shape chirality. Beyond intensity, chirality is also encoded in the polarization of the SH-scattered light.

Figure 3a shows a diagram of right-angled SH chiroptical scattering, in the dual circularly polarized light configuration. The setup now comprises a rotating quarter-wave plate and an analyzer, to resolve the circular polarization state of the SH light. Four distinct input–output circular polarization configurations are thus accessible: RCP_IN_-RCP_OUT_, LCP_IN_-LCP_OUT_, RCP_IN_-LCP_OUT_, and LCP_IN_-RCP_OUT_, see Figure 3b. In turn, these enable two separate chiroptical observables, akin to the Nafie’s degrees of circular polarization that are well-established in Raman optical activity.^50^

**Figure 3:**
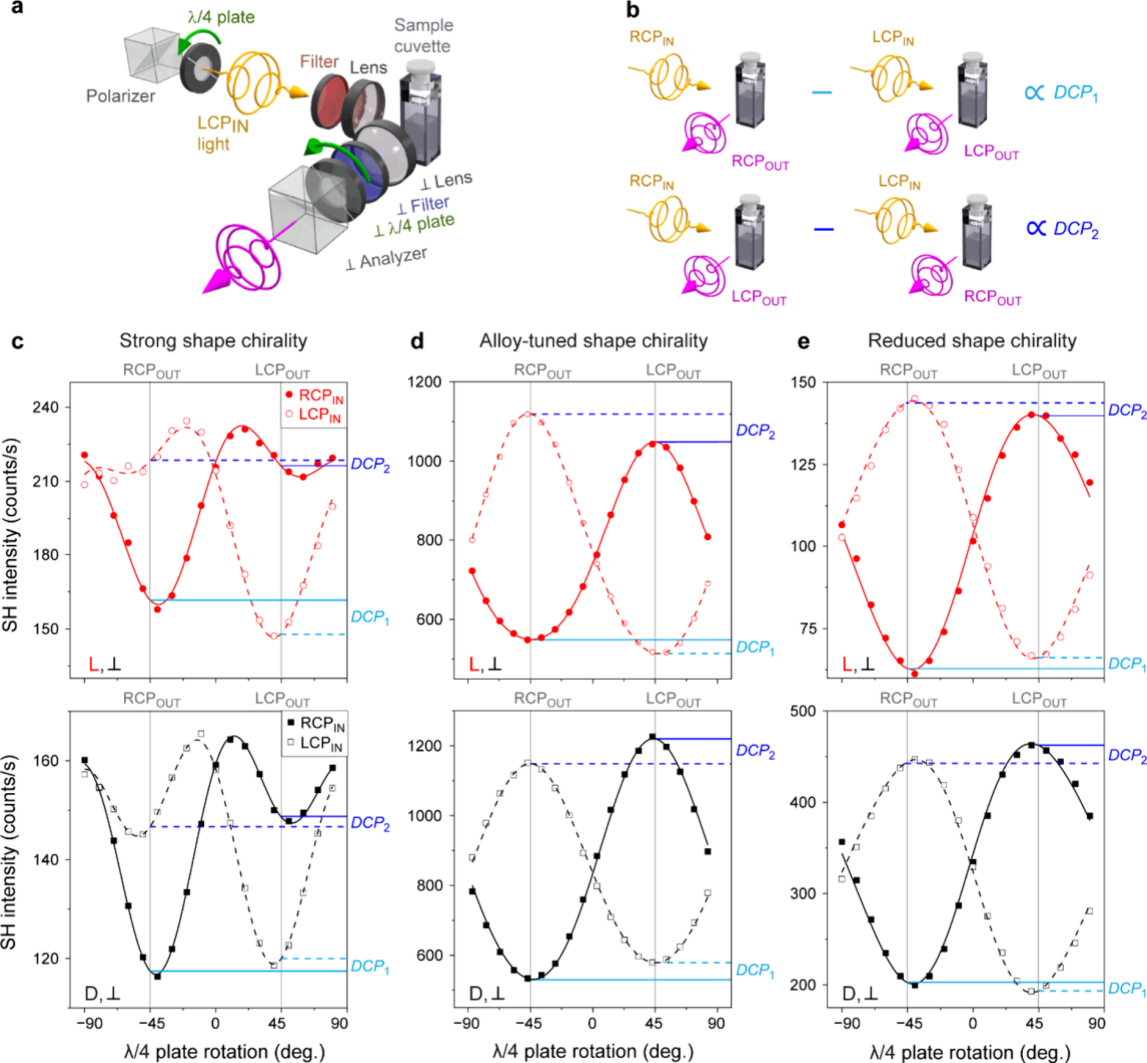
Robust dual-circular polarization signatures in SH right-angled (⊥) scattering despite reduced shape chirality. **a**, Schematic of the measurement geometry. **b**, The four circular polarizer–analyzer combinations defining *DCP*_1_ and *DCP*_2_ (eqs 2–3). **c–e**, SH intensity versus analyzer rotation angle for nanocrystals with strong shape chirality (c), alloy-tuned chirality (d), and reduced chirality (e). Filled/open symbols denote RCP_IN_/LCP_IN_. Solid/dashed lines are Stokes fits. Opposite signs of *DCP*_1_ and *DCP*_2_ are observed for L and D enantiomorphs.

Here, we define the normalized degrees of circular polarization as

2 $$DCP_{1}=\frac{I_{{\mathrm{RCP}}_{\mathrm{IN}}-{\mathrm{RCP}}_{\mathrm{OUT}}}^{\left(2\omega \right)}-I_{{\mathrm{LCP}}_{\mathrm{IN}}-{\mathrm{LCP}}_{\mathrm{OUT}}}^{\left(2\omega \right)}}{I_{{\mathrm{RCP}}_{\mathrm{IN}}-{\mathrm{RCP}}_{\mathrm{OUT}}}^{\left(2\omega \right)}+I_{{\mathrm{LCP}}_{\mathrm{IN}}-{\mathrm{LCP}}_{\mathrm{OUT}}}^{\left(2\omega \right)}}$$

and

3 $$DCP_{2}=\frac{I_{{\mathrm{RCP}}_{\mathrm{IN}}-{\mathrm{LCP}}_{\mathrm{OUT}}}^{\left(2\omega \right)}-I_{{\mathrm{LCP}}_{\mathrm{IN}}-{\mathrm{RCP}}_{\mathrm{OUT}}}^{\left(2\omega \right)}}{I_{{\mathrm{RCP}}_{\mathrm{IN}}-{\mathrm{LCP}}_{\mathrm{OUT}}}^{\left(2\omega \right)}+I_{{\mathrm{LCP}}_{\mathrm{IN}}-{\mathrm{RCP}}_{\mathrm{OUT}}}^{\left(2\omega \right)}}$$

In Figure 3c-e, for the three types of samples under study, the upper panels, in red, correspond to the L enantiomorphs and the lower panels, in black, correspond to the D enantiomorphs. These figures show the SH intensity recorded as a function of the analyzing quarter-wave plate rotation angle. At 0° the quarter-wave plate’s fast axis is along the direction of the analyzer. Thus, the vertical lines at −45° and +45° correspond to RCP_OUT_ and LCP_OUT_, respectively. The data points with solid and empty circles denote RCP_IN_ and LCP_IN_, respectively. These data points are fitted to Stokes parameters, and the fits are represented with full and dashed lines.

Moreover, in Figure 3c-e, the intercepts between data fitting lines and horizontal lines correspond to the four polarizer-analyzer combinations in Figure 3b. The SH intensities for these combinations are indicated with horizontal lines that allow us to calculate *DCP*_1_ (cyan) and *DCP*_2_ (dark blue). These values are addressed in the discussion section.

Forward-scattered SH light exhibits analogous but geometry-dependent dual-circular signatures (Figure 4). The polarization analysis protocol is identical to that used in right-angled detection, but SH light is now collected in the forward (∥) direction, see Figure 4a,b.

**Figure 4:**
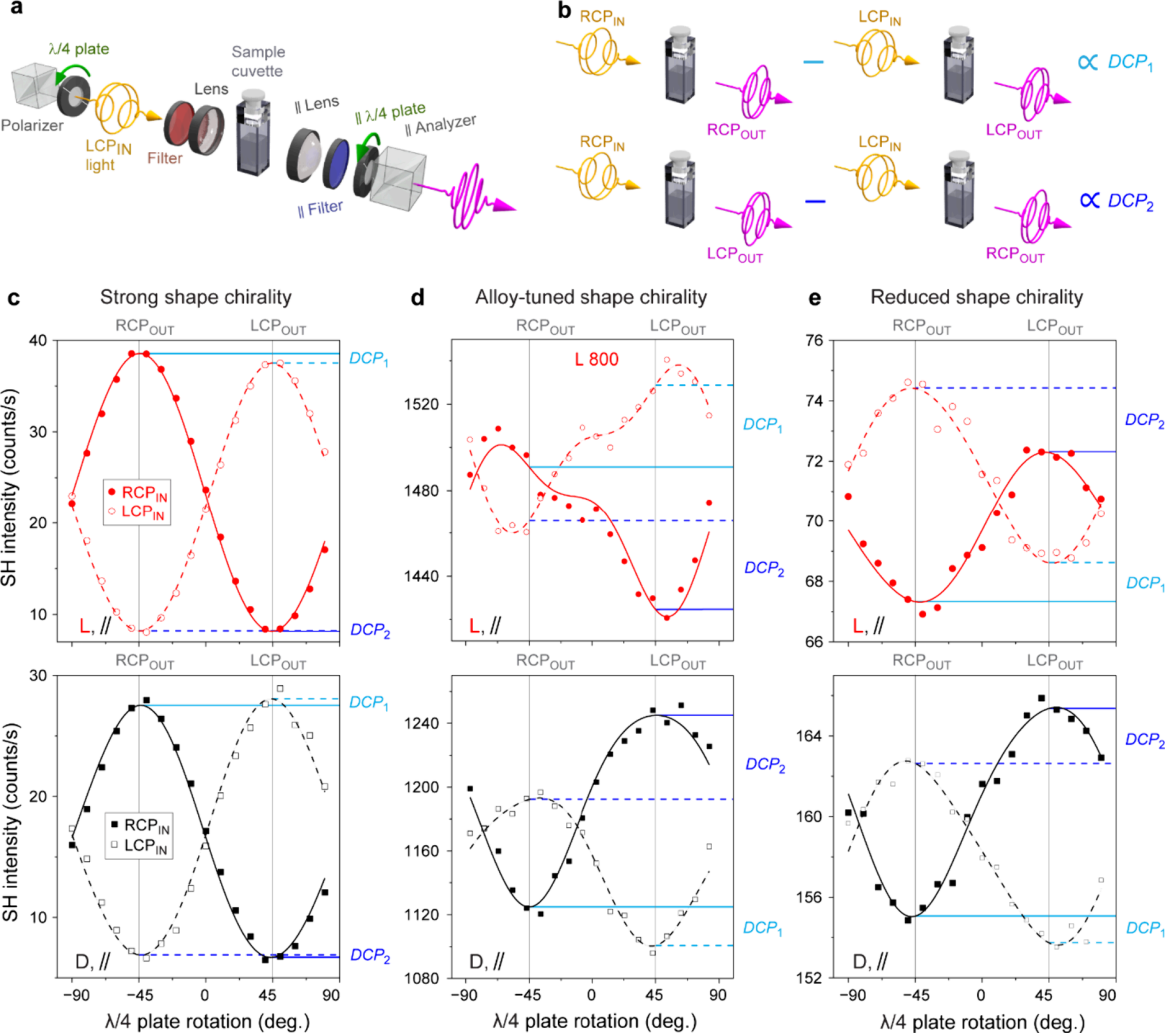
Robust dual-circular polarization signatures in SH forward (∥) scattering despite reduced shape chirality. **a**, Schematic of the forward-detected SH chiroptical scattering experiment. **b**, Circular polarizer–analyzer combinations used to extract the normalized observables *DCP*_1_ and *DCP*_2_ (eqs 2–3). **c–e**, SH intensity versus analyzer quarter-wave plate angle for nanocrystals with strong shape chirality (c), alloy-tuned chirality (d), and reduced chirality (e). Top panels correspond to L enantiomorphs and bottom panels to D enantiomorphs. Filled/open symbols denote RCP_IN_ /LCP_IN_; solid/dashed lines are Stokes fits. Nonzero *DCP*_1_ and *DCP*_2_ are observed for all samples and reverse sign between enantiomorphs.

As in the ∥ geometry, the SH intensity varies sinusoidally with analyzer quarter-wave plate angle and is well-described by Stokes-parameter fits (Figure 4c–e). The extracted circular components at −45° and +45° yield nonzero *DCP*_1_ and *DCP*_2_ for all three sample types. Importantly, both observables reverse sign between L and D enantiomorphs, confirming the chiral origin of the signal.

While the qualitative behavior mirrors that observed in ⊥ scattering, the relative magnitudes of *DCP*_1_ and *DCP*_2_ differ systematically between geometries. This geometry dependence indicates that the balance between dominant achiral and weaker chiral nonlinear tensor elements is detection-direction dependent, consistent with interference between multipolar radiation channels. Notably, even for nanocrystals with reduced shape chirality, both *DCP* observables remain clearly resolved in the forward direction.

Table 1 presents all of the chiroptical observables extracted from our results. We note that these observables consistently reverse sign between enantiomers and that the magnitudes vary.

**Table 1:** Nonlinear Chiroptical Parameters Extracted from Figures 2–4 ^a^

	Strong shape		Alloy-tuned		Reduced	
Measure	**L**	**D**	**L**	**D**	**L**	**D**
(*g*_NL_)_⊥_	0.010	–0.015	–0.029	0.020	–0.050	0.022
(*g*_NL_)_∥_	0.027	–0.019	–0.028	0.032	–0.030	0.016
*DCP*_1⊥_	0.045	–0.013	0.033	–0.042	–0.023	0.023
*DCP*_2⊥_	–0.005	0.007	–0.032	0.030	–0.014	0.020
*DCP*_1∥_	0.014	–0.011	–0.013	0.011	–0.010	0.005
*DCP*_2∥_	0.000	–0.015	–0.014	0.022	–0.014	0.009

a All SH-based observables show enantiomorph-dependent sign reversal, or near-sign reversal, across the three sample classes.

To aid interpretation of the polarization-resolved observables, we introduce here a reduced interference-based description, while a more complete symmetry-based tensor framework is developed in the Supporting Information. Generally, for chiral systems, the SH intensity can be expressed as

4 $$I\left(2\omega \right)\propto {\left|\chi_{eff}^{achiral}\pm \chi_{eff}^{chiral}\right|}^{2}$$

where the effective nonlinear susceptibility components (χ) are defined by their behavior under mirror inversion: the achiral term is invariant, whereas the chiral term reverses sign between enantiomorphs. Expanding this expression shows that SH chiroptical observables arise from interference between these two contributions. Accordingly, the nonlinear dissymmetry (or g-factor) scales as

5 $$g_{\mathrm{NL}}\propto \frac{\left|\chi_{eff}^{chiral}\right|\left|\chi_{eff}^{achiral}\right|}{{\left|\chi_{eff}^{chiral}\right|}^{2}+{\left|\chi_{eff}^{achiral}\right|}^{2}}\cos\varphi \approx \frac{\left|\chi_{eff}^{chiral}\right|}{\left|\chi_{eff}^{achiral}\right|}\cos\varphi ,\mathrm{for}\;\left|\chi_{eff}^{chiral}\right|\ll \left|\chi_{eff}^{achiral}\right|$$

where φ is the phase difference between the mirror-even and mirror-odd components. This relative phase is expected to depend on resonance conditions, local-field effects, morphology, and detection geometry and can therefore enhance, suppress, or invert the measured chiroptical contrast. Thus, to leading order, the SH chiroptical contrast reflects the experimentally projected balance between mirror symmetry-even and mirror symmetry-odd nonlinear response channels.

The same interference principle governs the polarization-resolved observables *DCP*_1_, *DCP*_2_, and g_NL_. Although their explicit forms differ, each quantity measures a geometry-dependent projection of the ratio |χ_*eff*_^*chiral*^|/|χ_*eff*_^*achiral*^|.

Importantly, these effective susceptibilities are not fixed material constants; rather, they are linear combinations of underlying tensor elements selected by the excitation polarization, detection geometry (⊥ or ∥), and analyzer configuration. Different experimental observables therefore interrogate different mixtures of mirror-even and mirror-odd tensor components. This naturally explains why *DCP*_1_ and *DCP*_2_ vary in magnitude between geometries, while consistently reversing sign between enantiomorphs.

This interference-based framework also clarifies why SH scattering retains strong handedness contrast even when linear circular dichroism is substantially reduced. In linear optics, CD typically represents a small chiral correction to a dominant electric-dipole response, so the observable scales against a large achiral background. In contrast, the SH observables depend explicitly on the ratio of chiral to achiral nonlinear contributions within the same order of response. Because polarization selection and detection geometry can suppress or redistribute the effective achiral component, the relative weight of the mirror-odd contribution can remain appreciable. Moreover, the phase factor cos φ introduces an additional lever: geometry-dependent phase relationships between tensor components can enhance or diminish the observed contrast.

Consequently, even in nanocrystals with markedly reduced shape chirality – for which the linear CD decreases by roughly an order of magnitude – the nonlinear observables g_NL_, *DCP*_1_, and *DCP*_2_ remain clearly measurable. The measured nonlinear chiroptical observables remain comparable in magnitude to values previously reported for chiroptical SH scattering from Ag, Au, CdTe, and Si nanohelices, despite the substantially weaker linear chiroptical response observed in the reduced-shape Te nanocrystals.^33–36^ Direct quantitative comparison remains difficult because the observables depend strongly on excitation wavelength, resonance conditions, morphology, and detection geometry. Differences in absolute SH count rates between the three sample classes are not interpreted directly, since our analysis focuses on normalized chiroptical observables (g_NL_, *DCP*_1_, *DCP*_2_), which compare polarization channels recorded under identical conditions and are therefore insensitive to overall signal scaling.

In summary, we have tracked chirality evolution within a single colloidal nanocrystal platform by comparing tellurium nanocrystals exhibiting strong, alloy-tuned, and reduced shape chiralities. While linear circular dichroism decreases markedly as morphological asymmetry is attenuated, polarization-resolved second-harmonic scattering retains clear handedness-dependent signatures across six independent observables and two detection geometries. The consistent sign reversal between enantiomorphs and the geometry-dependent magnitudes of the nonlinear responses are well-described by interference between mirror-even and mirror-odd susceptibility components.

By examining relative changes rather than absolute signal magnitudes, this study demonstrates that nonlinear chiroptical scattering remains sensitive to diminishing structural asymmetry even when the linear chiroptical contrast becomes weak. These results extend chiroptical harmonic scattering to nanocrystals exhibiting coupled crystal and shape chirality and establish a framework for tracking the symmetry evolution in liquid-phase nanomaterials. More broadly, these results demonstrate that polarization-resolved nonlinear scattering provides a robust route for detecting and monitoring weak structural asymmetry in liquid-phase nanomaterials with consistent signatures across six independent observables.

## Supplementary Material



## Data Availability

The data that support the findings of this study are openly available in the repository of the University of Bath at 10.15125/BATH-01658.

## Notes

Theoretical model for polarization-resolved second-harmonic scattering; derivation of the nonlinear observables g_NL_, *DCP*_1_, *DCP*_2_; symmetry-based tensor-interference framework; experimental procedures for nanocrystal synthesis and optical characterization; additional statistical analysis of the nonlinear measurements (PDF)
