# Supplementary material for: Tracking Chirality Evolution in Tellurium Nanocrystals Via Polarization-Resolved Second-Harmonic Scattering
Source: Nano Lett. 2026 Jun 15;26(26):8497–504. doi: 10.1021/acs.nanolett.6c01271 (PMC13352940; doi:10.1021/acs.nanolett.6c01271)
Supplement: Supplementary file 1 [file nl6c01271_si_001.pdf]

Tracking Chirality Evolution in Tellurium Nanocrystals via Polarization-Resolved Second-Harmonic Scattering

*Ruidong Ji, Bar Reuven, Bradleigh Kerrigan, Gil Markovich, and Ventsislav K. Valev**

R. Ji, B. Kerrigan
Department of Physics, University of Bath, Bath, BA2 7AY, UK.

B. Reuven, G. Markovich
School of Chemistry, the Raymond and Beverly Sackler Faculty of Exact Sciences, Tel Aviv University, Tel Aviv 6997801, Israel

V. K. Valev
Department of Physics, University of Bath, Bath, BA2 7AY, UK.
Department of Electronic & Electrical Engineering, University of Bath, Bath, BA2 7AY, UK

E-mail: v.k.valev@bath.ac.uk

Keywords: Chirality, chirality transfer, nanoparticles, nonlinear optics, chiroptical effects

Theoretical model section

To clarify the physical origin of the nonlinear chiroptical observables g_{NL}, DCP_1, and DCP_2, we introduce a reduced symmetry-based model for polarization-resolved second-harmonic (SH) scattering. The aim is to identify the minimal ingredients required to generate handedness-dependent SH contrast.

A full microscopic description would involve many independent complex tensor elements, orientational averaging over arbitrary particle geometries, multipolar radiation channels, spatially varying local-field enhancements, spectral dispersion, and detailed nanocrystal–solvent boundary conditions. Here we retain only the dominant symmetry contributions. Each nanocrystal is represented by a single privileged structural axis, and each nonlinear susceptibility tensor is reduced to one effective longitudinal component defined along this axis. Within this reduced framework, crystal chirality and morphology-derived chirality contribute collectively through effective mirror symmetry-odd nonlinear response channels. Orientational averaging is treated through direction-cosine averages, while local-field effects are incorporated phenomenologically through scalar enhancement factors. In the present minimal model these factors are treated as scalar quantities for analytical transparency; introducing tensorial or complex local-field coupling would generate additional polarization-dependent interference terms and stronger resonance sensitivity.

Within this simplified model, the measured SH observables emerge naturally from interference between mirror symmetry-even (those that keep their sign under mirror symmetry reflection) and mirror symmetry-odd (those that change sign under mirror symmetry reflection) mathematical terms. The resulting expressions reproduce the key qualitative features observed experimentally: sign reversal between enantiomorphs, geometry-dependent polarization responses, and the persistence of nonlinear chiroptical contrast even when linear circular dichroism becomes weak.

We write the second-order electric- and magnetic-dipoles generated at the second-harmonic in terms of the *local* fundamental fields $\mathbf{E}_{loc}(\omega)$ and $\mathbf{B}_{loc}(\omega)$ (frequency arguments omitted for

compactness). The expressions for the second-harmonic electric- ($\mathbf{p}$) and magnetic-dipoles ($\mathbf{m}$) are then:

$$\begin{aligned}\mathbf{p}(2\omega) &= \boldsymbol{\beta}_{\boldsymbol{EE}}\mathbf{E}_{loc}\mathbf{E}_{loc} + \boldsymbol{\chi}_{\boldsymbol{EB}}\mathbf{E}_{loc}\mathbf{B}_{loc} + i\mathbf{G}_{\boldsymbol{BB}}\mathbf{B}_{loc}\mathbf{B}_{loc} \\ \mathbf{m}(2\omega) &= \boldsymbol{\mu}_{\boldsymbol{BB}}\mathbf{B}_{loc}\mathbf{B}_{loc} + \boldsymbol{\chi}'_{\boldsymbol{EB}}\mathbf{E}_{loc}\mathbf{B}_{loc} + i\mathbf{G}'_{\boldsymbol{EE}}\mathbf{E}_{loc}\mathbf{E}_{loc}\end{aligned} \qquad \text{(SI-1)}$$

where $\boldsymbol{\beta}_{\boldsymbol{EE}}$ is the second-order hyperpolarizability tensor, $\boldsymbol{\mu}_{\boldsymbol{BB}}$ is the magnetic hyperpolarizability tensor, $\mathbf{G}_{\boldsymbol{BB}}$ and $\mathbf{G}'_{\boldsymbol{EE}}$ are the electro-magnetic cross coupling hyperpolarizability tensors, and $\boldsymbol{\chi}_{\boldsymbol{EB}}$, $\boldsymbol{\chi}'_{\boldsymbol{EB}}$ are the mixed-term hyperpolarizability tensors.

While magnetic-field–driven nonlinearities are typically neglected in molecular nonlinear optics, they are retained here to account for strong magnetic near-fields and retardation effects in nanoscale chiral scatterers, where magnetic responses can be significant.

For an ensemble of randomly oriented uniaxial scatterers, the effective second-order response is often dominated by a single largest term, e.g. this is often the β_{zzz} component for the electric-dipole hyperpolarizability. We therefore chose to represent each second-order hyperpolarizability tensor by a single independent scalar component, corresponding to the longitudinal responses relative to the main axis of the scatterer. This component will be oriented along the vector $\hat{\mathbf{u}}$, designating the main axis of the scatterer $\hat{\mathbf{u}} = (l_x, l_y, l_z)$, where l_i are the direction cosines and where $\hat{\boldsymbol{x}}$, $\hat{\boldsymbol{y}}$, $\hat{\boldsymbol{z}}$, are the laboratory frame axes. The corresponding tensor elements are $\beta_{\parallel EE}$, $\mu_{\parallel BB}$, $G_{\parallel BB}$, $G'_{\parallel EE}$, $\chi_{\parallel EB}$ and $\chi'_{\parallel EB}$.

Local-field effects are included phenomenologically via frequency-dependent local-field factors; no specific microscopic model is assumed, hence $\mathbf{E}_{loc} = L_{\omega}\mathbf{E}_{in}$ and $\mathbf{B}_{loc} = L_{\mathrm{m}\omega}\mathbf{B}_{in}$.

Using $\mathbf{B}_{in} = \frac{1}{\omega}\mathbf{k}_{in} \times \mathbf{E}_{in}$ to fix the directions of the fields, we choose $\mathbf{k}_{in}$ to be along $\hat{\boldsymbol{z}}$. We retain the magnetic local-field factors for generality; in the weak-retardation limit they reduce to unity. The second-harmonic electric-dipole is decomposed as:

$$\mathbf{p}(2\omega) = L_{\omega}^{2}\beta_{\parallel EE}(\mathbf{E}_{in}\cdot\hat{\mathbf{u}})^{2}\hat{\mathbf{u}} + L_{\omega}L_{\mathrm{m}\omega}\chi_{\parallel EB}(\mathbf{E}_{in}\cdot\hat{\mathbf{u}})(\mathbf{B}_{in}\cdot\hat{\mathbf{u}})\hat{\mathbf{u}} + iL_{m\omega}^{2}G_{\parallel BB}(\mathbf{B}_{in}\cdot\hat{\mathbf{u}})^{2}\hat{\mathbf{u}}. \qquad \text{(SI-2)}$$

Similarly, the magnetic-dipole is written as:

$$\mathbf{m}(2\omega) = L_{m\omega}^{2}\mu_{\parallel BB}(\mathbf{B}_{in}\cdot\hat{\mathbf{u}})^{2}\hat{\mathbf{u}} + L_{\omega}L_{\mathrm{m}\omega}\chi'_{\parallel EB}(\mathbf{E}_{in}\cdot\hat{\mathbf{u}})(\mathbf{B}_{in}\cdot\hat{\mathbf{u}})\hat{\mathbf{u}} + iL_{\omega}^{2}G'_{\parallel EE}(\mathbf{E}_{in}\cdot\hat{\mathbf{u}})^{2}\hat{\mathbf{u}}. \qquad \text{(SI-3)}$$

For analytical transparency, the effective coefficients are treated as real-valued within the present reduced model. Allowing complex-valued tensor elements would introduce additional relative phase factors between mirror symmetry-even and mirror symmetry-odd contributions, modifying the magnitude and potentially the sign of the resulting interference observables without altering the underlying symmetry arguments.

For left- and right-handed circularly polarized incident light $\mathbf{E}_{in}^{(\pm)} = \mathrm{E}_0\frac{1}{\sqrt{2}}(\hat{\mathbf{x}} \pm i\hat{\mathbf{y}})$ and $\mathbf{B}_{in}^{(\pm)}(\omega) = \mathrm{B}_0\frac{1}{\sqrt{2}}(\mp i\hat{\mathbf{x}} + \hat{\mathbf{y}})$. Their projections onto $\hat{\mathbf{u}}$ are indicated as $(\mathbf{E}_{in}\cdot\hat{\mathbf{u}})$ and $(\mathbf{B}_{in}\cdot\hat{\mathbf{u}})$.

The corresponding scattered SH fields are then obtained as:

$$\begin{aligned}\mathbf{E}_{sc}^{el}(2\omega,\hat{\mathbf{r}})_{local} &= C_{2\omega}\,\hat{\mathbf{r}} \times [\hat{\mathbf{r}} \times \mathbf{p}(2\omega)] \\ \mathbf{E}_{sc}^{mag}(2\omega,\hat{\mathbf{r}})_{local} &= C_{2\omega}\,\hat{\mathbf{r}} \times \mathbf{m}(2\omega)\end{aligned} \qquad \text{(SI-4)}$$

with $C_{2\omega} = \frac{k_{2\omega}^{2}}{4\pi\varepsilon_0}\frac{e^{ik_{2\omega}r}}{r}$.

In our experimental geometry, we consider the direction of observation as $\hat{\mathbf{r}} = [0, \sin(\theta), \cos(\theta)]$. The far-field is then obtained with the second-harmonic local field factors

$$\mathbf{E}_{sc}^{el}(2\omega,\hat{\mathbf{r}}) = L_{2\omega}\mathbf{E}_{sc}^{el}(2\omega,\hat{\mathbf{r}})_{local} = L_{2\omega}C_{2\omega}\,\hat{\mathbf{r}} \times [\hat{\mathbf{r}} \times \mathbf{p}(2\omega)]$$
$$\mathbf{E}_{sc}^{mag}(2\omega,\hat{\mathbf{r}}) = L_{\mathrm{m}2\omega}\mathbf{E}_{sc}^{mag}(2\omega,\hat{\mathbf{r}})_{local} = L_{\mathrm{m}2\omega}C_{2\omega}\,\hat{\mathbf{r}} \times \mathbf{m}(2\omega) \quad \text{(SI-5)}$$

We can then calculate $\mathbf{E}_{sc} = \mathbf{E}_{sc}^{el} + \mathbf{E}_{sc}^{mag}$ and $I_{sc}^{\pm}(2\omega) = \langle \mathbf{E}_{sc}^{\pm}\mathbf{E}_{sc}^{\pm *}\rangle$. Which leads to

$$g_{NL} = \frac{I_{sc}^{+}(2\omega) - I_{sc}^{-}(2\omega)}{I_{sc}^{+}(2\omega) + I_{sc}^{-}(2\omega)}. \quad \text{(SI-6)}$$

The sign of the g_{NL} is determined by:

$$I_{sc}^{+}(2\omega) - I_{sc}^{-}(2\omega) = 2L_{\omega}L_{\mathrm{m}\omega}\left(E_0{}^2 L_{\mathrm{m}2\omega}{}^2 L_{\omega}{}^2 G'_{\parallel EE}\chi'_{\parallel EB} - B_0{}^2 L_{2\omega}{}^2 L_{\mathrm{m}\omega}{}^2 G_{\parallel BB}\chi_{\parallel EB}\right)B_0E_0, \quad \text{(SI-7)}$$

The polarization state of the scattered SH field can be analysed by introducing directions for orienting the analyser. A convenient choice of transverse basis vectors is

$$\hat{\mathbf{e}}_1 = \hat{\mathbf{x}},\ \hat{\mathbf{e}}_2 = [0, \cos(\theta), -\sin(\theta)] \quad \text{(SI-8)}$$

The complex field amplitudes detected in the laboratory basis are obtained by projection:

$$\mathrm{E}_1 = \mathbf{E}_{sc}^{\pm} \cdot \hat{\mathbf{e}}_1,\ \mathrm{E}_2 = \mathbf{E}_{sc}^{\pm} \cdot \hat{\mathbf{e}}_2\ . \quad \text{(SI-9)}$$

And $\mathbf{E}_{sc}^{\pm} = \mathrm{E}_1^{\pm}\hat{\mathbf{e}}_1 + \mathrm{E}_2^{\pm}\hat{\mathbf{e}}_2$ represents a decomposition of the scattered field on the basis of the analyser directions.

We can then define the outgoing field amplitudes: $\hat{\mathbf{e}}_{LCP_{OUT}} = \frac{\hat{\mathbf{e}}_1 + i\hat{\mathbf{e}}_2}{\sqrt{2}}$, and $\hat{\mathbf{e}}_{RCP_{OUT}} = \frac{\hat{\mathbf{e}}_1 - i\hat{\mathbf{e}}_2}{\sqrt{2}}$

Then $\mathbf{E}_{LCP_{OUT}}^{\pm} = \mathbf{E}_{sc}^{\pm} \cdot \hat{\mathbf{e}}_{LCP_{OUT}} = \frac{\mathrm{E}_1 + i\mathrm{E}_2}{\sqrt{2}}$, and $\mathbf{E}_{RCP_{OUT}}^{\pm} = \mathbf{E}_{sc}^{\pm} \cdot \hat{\mathbf{e}}_{RCP_{OUT}} = \frac{\mathrm{E}_1 - i\mathrm{E}_2}{\sqrt{2}}$.

This leads to measured intensities:

$$I_{LCP_{OUT}}^{\pm}(2\omega) = \left\langle \mathbf{E}_{LCP_{OUT}}^{\pm}\mathbf{E}_{LCP_{OUT}}^{\pm *}\right\rangle;\ I_{RCP_{OUT}}^{\pm}(2\omega) = \left\langle \mathbf{E}_{RCP_{OUT}}^{\pm}\mathbf{E}_{RCP_{OUT}}^{\pm *}\right\rangle \quad \text{(SI-10)}$$

We can then define DCP_1 as:

$$DCP_1 = \frac{I_{LCP_{OUT}}^{+}(2\omega) - I_{RCP_{OUT}}^{-}(2\omega)}{I_{LCP_{OUT}}^{+}(2\omega) + I_{RCP_{OUT}}^{-}(2\omega)}, \quad \text{(SI-11)}$$

And DCP_2 as:

$$DCP_2 = \frac{I_{RCP_{OUT}}^{+}(2\omega) - I_{LCP_{OUT}}^{-}(2\omega)}{I_{RCP_{OUT}}^{+}(2\omega) + I_{LCP_{OUT}}^{-}(2\omega)}. \quad \text{(SI-12)}$$

To shorten the notations, we can define:

$$\mathrm{A2}_E = \frac{1}{2}E_0^2 L_{2\omega}C_{2\omega}L_{\omega}^2\beta_{\parallel} \qquad \mathrm{A2}_G = \frac{1}{2}B_0^2 L_{2\omega}C_{2\omega}L_{m\omega}^2 G_{\parallel 2\omega} \qquad \mathrm{A2}_{EB} = \frac{1}{2}\mathrm{E}_0\mathrm{B}_0 L_{2\omega}C_{2\omega}L_{\omega}L_{\mathrm{m}\omega}\chi_{\parallel}$$
$$\mathrm{B2}_M = \frac{1}{2}B_0^2 L_{\mathrm{m},2\omega}C_{2\omega}L_{m\omega}^2\mu_{\parallel 2\omega} \qquad \mathrm{B2}_G = \frac{1}{2}E_0^2 L_{\mathrm{m},2\omega}C_{2\omega}L_{\omega}^2 G'_{\parallel 2\omega} \qquad \mathrm{B2}_{EB} = \frac{1}{2}\mathrm{E}_0\mathrm{B}_0 L_{2\omega}C_{2\omega}L_{\omega}L_{\mathrm{m}\omega}\chi'_{\parallel} \quad \text{(SI-13)}$$

with $C_{2\omega} = \frac{k_{2\omega}^2}{4\pi\varepsilon_0}\frac{e^{ik_{2\omega}r}}{r}$.

Of these, the mirror symmetry-even are $\mathrm{A2_{EB}}$, $B2_G$, $B2_M$ and the mirror symmetry-odd are $\mathrm{A2}_E$, $\mathrm{A2}_G$ and $\mathrm{B2_{EB}}$. After orientational averaging, the resulting SH intensities contain interference terms between mirror symmetry-even and mirror symmetry-odd nonlinear contributions. These terms are responsible for the handedness-dependent observables measured experimentally. Specifically, we obtain:

$$g_{NL} = -\left(\frac{2\mathrm{B2}_{\mathrm{EB}}\mathrm{B2}_G - 2\mathrm{A2}_{\mathrm{EB}}\mathrm{A2}_G}{\mathrm{B2}_M{}^2 + \mathrm{B2}_G{}^2 + \mathrm{B2\_EB}^2 + \mathrm{A2}_G{}^2 + \mathrm{A2\_EB}^2 + \mathrm{A2}_E{}^2}\right) . \quad \text{(SI-14)}$$

Importantly, the observables g_{NL}, DCP_1, and DCP_2 do not constitute intrinsic scalar measures of chirality. Rather, they represent geometry-dependent interference observables constructed from specific projections of mirror symmetry-even and mirror symmetry-odd nonlinear tensor contributions. Consequently, changes in excitation wavelength, local-field enhancement, morphology, orientational averaging, resonance conditions, or detection geometry can modify both the magnitude and sign of the measured response without implying a change in the underlying handedness itself. Different polarization observables and detection geometries therefore probe different effective tensor combinations and interference pathways, such that forward and right-angled scattering need not exhibit identical chiroptical responses.

Equation SI-14 shows explicitly that g_{NL} originates from products of mirror symmetry-even and mirror symmetry-odd tensor contributions weighted by local-field factors. The observable therefore depends on tensor projections and orientational averaging, rather than representing an intrinsic scalar measure of chirality.

The numerator for DCP_1 is: $\Delta I_{CC} = \frac{32}{35}(\mathrm{A2}_G\mathrm{B2}_M + (\mathrm{B2}_{\mathrm{EB}} - \mathrm{A2}_E)\mathrm{B2}_G - \mathrm{A2}_{\mathrm{EB}}\mathrm{A2}_G)$; it changes sign under mirror inversion.

The numerator for DCP_2 is: $\Delta I_{CR} = \frac{32}{35}(-\mathrm{A2}_G\mathrm{B2}_M + (\mathrm{B2}_{\mathrm{EB}} + \mathrm{A2}_E)\mathrm{B2}_G - \mathrm{A2}_{\mathrm{EB}}\mathrm{A2}_G)$; it changes sign under mirror inversion.

We therefore see that the difference between DCP_1 and DCP_2 arises from the signs and relative weighting of the contributing tensorial interference terms.

Although highly simplified, this reduced model captures the central physical mechanism underlying the experimental observations: nonlinear chiroptical scattering arises from interference between mirror symmetry-even and mirror symmetry-odd response channels whose relative weighting depends on excitation polarization, detection geometry, local-field enhancement, and orientational averaging. Consequently, observables such as g_{NL}, DCP_1, and DCP_2 remain sensitive to weak structural asymmetry even when linear chiroptical contrast becomes substantially reduced, which is consistent with the experimental observations reported in the main text.

Experimental section

Growth of Te nanocrystals: Te nanocrystals were synthesized by the aqueous reduction of sodium tellurite (Na2TeO3) using hydrazine and L- or D-penicillamine. In a 20 mL scintillation vial under constant stirring, the following were added in the sequential order: 0.5 mL ultrapure water, 1 mL Na2TeO3 0.1 M, 50 μL NaOH 1 M, and 1 mL L- or D-penicillamine 0.1 M. After 3 minutes of stirring, 2.5 mL hydrazine hydrate (50 – 60%) was added to initiate the reaction. For nanocrystals exhibiting strong shape chirality, the reaction was allowed to proceed for 15 minutes.
Se-alloyed Te nanocrystals were prepared in a similar manner. Immediately after the addition of hydrazine, 35 μL of 0.1 M selenourea dissolved in dimethylformamide was injected to the reaction mixture (corresponding to ~3.5% at. nominal Se). The reaction was then allowed to proceed for 30 minutes.

To produce Te nanocrystals with reduced shape chirality, the standard synthesis (using L- or D-penicillamine) was carried out for 60 minutes at ambient temperature. Then, the reaction

temperature was increased to 40 °C and maintained for an additional 3 hours to facilitate further crystal growth and morphological transformation.

All reactions were terminated by diluting the mixtures ×2 with a 0.1 M solution of sodium dodecyl sulfate (SDS), followed by centrifugation at 5000 RCF for 10 minutes. The precipitate was finally redispersed in ~15 mL SDS 0.05 M for further storage and characterization.

Scanning Electron Microscopy: The samples for scanning electron microscopy were prepared by three consecutive centrifugation cycles, redispersing the particles in ultrapure water and drop-casting. The morphology of the nanocrystals was imaged by a Gemini 300 scanning electron microscope (Zeiss, Germany) operated at 5 kV and recording the secondary electrons signal.

CD spectroscopy: Linear CD measurements were obtained using a Chirascan CD spectrometer (Applied Photophysics, UK) at 250 to 800 nm with 3 nm bandwidth and 5 nm sampling intervals.

Nonlinear chiroptical characterization: The excitation beam was generated by an optical parametric oscillator (OPO) pumped by an 80 MHz Ti:Sapphire laser and tuned to 1080 nm. The incident power (0–60 mW, 10 steps) was controlled using a half-wave plate and a Glan–laser polarizer, while the handedness of circular polarization light (CPL) was adjusted with a super-achromatic quarter-wave plate. A 1000 nm long-pass filter was used to remove residual second harmonic (SH) scattering light before the beam was focused into the cuvette with sample by an achromatic doublet lens. Scattered light was collected in both forward and right-angled directions. Residual fundamental light was filtered with bandpass coloured glass filters, and the SH scattering signal was further selected with a 540 nm (10 nm bandwidth) bandpass filter before detection by a PMT. The signal was processed with a pre-amplifier and photon counter synchronized to an optical chopper.

For the power-dependent measurements, the SH scattering intensity at each power was recorded 12 times before switching the handedness of the incident CPL. The power was increased stepwise up to the maximum value, and this full power scan was repeated at least 10 times for each sample.

For polarization analysis, a quarter-wave plate combined with a polarizer was placed after the collection lens in the right-angled and forward directions. The incident power was kept constant. The quarter-wave plate was rotated from –90° to 80° in 10° steps relative to the transmission axis of the subsequent polarizer, whose transmission polarization direction was fixed vertically. The handedness of the incident CPL was switched after completing one full rotation cycle. At each angle, 4 measurements were taken. The right-angled and forward polarization scans were repeated at least 12 times for each sample.
